# Cryostructuring of Polymeric Systems. 49. Unexpected “Kosmotropic-Like” Impact of Organic Chaotropes on Freeze–Thaw-Induced Gelation of PVA in DMSO

**DOI:** 10.3390/gels4040081

**Published:** 2018-10-08

**Authors:** Vladimir I. Lozinsky, Olga Yu. Kolosova, Dmitrii A. Michurov, Alexander S. Dubovik, Viktor G. Vasil’ev, Valerij Ya. Grinberg

**Affiliations:** A. N. Nesmeyanov Institute of Organoelement Compounds, Russian Academy of Sciences, ul. Vavilova 28, Moscow 119991, Russian Federation; kolosova@ineos.ac.ru (O.Y.K.); dmitriial7.8@gmail.com (D.A.M.); adubovik@ineos.ac.ru (A.S.D.); viktor@ineos.ac.ru (V.G.V.); grinberg@ineos.ac.ru (V.Y.G.)

**Keywords:** poly(vinyl alcohol) cryogels, dimethyl sulfoxide, chaotropes, kosmotropes, unexpected effect

## Abstract

Urea (URE) and guanidine hydrochloride (GHC) possessing strong chaotropic properties in aqueous media were added to DMSO solutions of poly(vinyl alcohol) (PVA) to be gelled via freeze–thaw processing. Unexpectedly, it turned out that in the case of the PVA cryotropic gel formation in DMSO medium, the URE and GHC additives caused the opposite effects to those observed in water, i.e., the formation of the PVA cryogels (PVACGs) was strengthened rather than inhibited. Our studies of this phenomenon showed that such “kosmotropic-like” effects were more pronounced for the PVACGs that were formed in DMSO in the presence of URE additives, with the effects being concentration-dependent. The additives also caused significant changes in the macroporous morphology of the cryogels; the commonly observed trend was a decrease in the structural regularity of the additive-containing samples compared to the additive-free gel sample. The viscosity measurements revealed consistent changes in the intrinsic viscosity, Huggins constant, and the excess activation heat of the viscosity caused by the additives. The results obtained evidently point to the urea-induced decrease in the solvation ability of DMSO with respect to PVA. As a result, this effect can be the key factor that is responsible for strengthening the structure formation upon the freeze–thaw gelation of this polymer in DMSO additionally containing additives such as urea, which is capable of competing with PVA for the solvent.

## 1. Introduction

Polymeric cryogels are macroporous gel materials that are formed as a result of the consecutive freezing of molecular or colloidal solutions of certain precursors that are potentially capable of gelling, subsequent incubation of the system in a frozen state, and then thawing [1,2,3,4,5]. The macroporosity of cryogels is generated by the polycrystals of the frozen solvent, while the gel formation itself proceeds within the volume of the remaining unfrozen inclusions (so-called unfrozen liquid microphase) [6] where the precursors are concentrated [1,5]. Similar to the classification of ‘conventional’ gels that are formed at temperatures above the freezing points of the gelling systems, cryogels can be categorized into three main types with the respect to the nature of interchain links, viz., covalent (chemically cross-linked), non-covalent (physical), and ionic/coordination cryogels [1]. Among all of the cryogel-type matrices, non-covalent cryogels based on poly(vinyl alcohol) (PVA) are most often studied as objects of basic and applied research [1,2,3,4,5,7,8,9,10,11]. This is first of all due to the simple preparation procedure of various PVA cryogels (PVACGs) where no additional cross-linking agents are required. Only the freezing–thawing of PVA solutions is enough to produce the final gel material. Second, PVA itself is a cheap and readily available macromolecular compound. Third, an amazingly wide range of applications of these gel matrices, from medicine [12,13,14,15,16,17,18,19,20], biotechnology [9,21,22,23,24,25,26,27,28], and food protection [29] to ecology [30] and construction in the cold climate regions [31,32] have attracted great interest.

The physicochemical properties and microstructure of PVACGs are known to depend on many factors. These include the molecular weight, the amount of residual O-acyl groups, the chain tacticity of the gelling polymer, the PVA concentration in its solution prior to the freeze–thaw gelation, the solvent used, the absence or the presence of other solutes, and, certainly, the conditions of cryogenic processing, namely, the rate of cooling upon the freezing of the polymer solutions, the temperature and duration of the freezing and frozen storage, the rate of heating upon defrosting the samples, and the number of freeze–thaw cycles (see reviews by Lozinsky et al., Gun’ko et al., Nambu, Peppas et al., Guitiérres et al. [1,2,5,7,8,9,10,11] and the references cited therein).

In most of the reported studies, water was used to prepare the PVA solutions to be gelled cryogenically. In these cases, the presence of soluble or insoluble (dispersed fillers) additives can have a significant effect on the PVACG formation *per se* and on the characteristics of the resultant cryogels. Since the interchain links in the nodes (microcrystallites [33,34,35,36]) of the three-dimensional (3D) supramolecular networks in the physical PVACGs are numerous H-bonds [1,9,10,37,38,39], the most pronounced influence is especially inherent in the solutes that are able to exert a strong impact on the PVA hydrogen bonding as well as on the solvation of the polymer. For instance, when the initial PVA solutions additionally contain salting-in electrolytes such as LiCl or NaSCN, it causes a pronounced weakening of the final cryogels, whereas the addition of salting-out electrolytes (NaF, KCl, NaOAc, or Na_2_SO_4_) induces a significant increase in the strength and heat endurance of PVACGs [40,41,42]. Recently [43], it was found that very similar effects with respect to the cryotropic gelation of aqueous PVA solutions are inherent in the organic substances that can inhibit or, in contrast, favor H-bonding, i.e., organic *chaotropes* and *kosmotropes*, respectively [44]. In particular, such chaotropic agents as urea (URE) or guanidine hydrochloride (GHC) caused a decrease in the elasticity modulus and fusion temperature of PVACGs prepared from aqueous PVA solutions containing these additives. In turn, the presence of kosmotropic agents, e.g., trehalose (THL) or hydroxyproline facilitated an increase in the rigidity and heat endurance of the resulting cryogels, starting with certain concentrations of the additives [43].

Along with the preparation of various PVACGs formed in aqueous media, cryogels produced by the freezing–thawing of dimethyl sulfoxide (DMSO) solutions [1,45,46,47,48,49] or DMSO-*d*_6_ solutions [50,51] of the polymer are also known. Since DMSO is a thermodynamically better solvent than water for high-deacylated PVA samples [52], i.e., the affinity of DMSO to this polymer is higher compared to the affinity in the water–PVA pair, the PVA gelation in DMSO occurs poorly. Therefore, the PVA/DMSO cryogels are always weaker and have lower fusion temperatures (*T*_f_) than the PVACGs prepared from aqueous solutions of the same polymer concentration [46,47].

In this regard, it was reasonable to anticipate an increase in the rigidity and heat endurance of PVA/DMSO-based cryogels in the presence of DMSO-soluble additives that behave similar to kosmotropes in the case of the freeze–thaw gelation of PVA in aqueous medium. Indeed, experiments using additives of the above-mentioned kosmotropes (e.g., THL) demonstrated an increase in the elasticity modulus and fusion temperature of the respective PVA/DMSO cryogels. However, when we simply wanted to ascertain that the organic chaotropes URE or GHC can act in DMSO in the same “weakening” fashion as in water, an unexpected “kosmotropic-like” impact of URE and GHC on the freeze–thaw-induced gelation of PVA was observed [53]. In this paper, we report and discuss the results of subsequent studies of the peculiar features and causes of the title phenomenon.

## 2. Results and Discussion

### 2.1. Influence of Chaotropic and Kosmotropic Additives on the Gel Strength and Heat Endurance of PVACGs Prepared in DMSO Medium

As indicated in the Introduction, our previous study [43] showed that organic chaotropes, namely, non-ionic URE and ionic GHC, that were dissolved in the aqueous feed solutions of PVA caused a progressive decrease in the *E* and *T*_f_ values of the PVACGs formed using the freeze–thaw gelation technique. The higher the concentration of the chaotropic agent, the lower the elasticity modulus and the fusion temperature of the respective cryogels. Particularly, at an initial PVA concentration of 100 g/L, it was possible to prepare gel samples only at additive concentrations of at most 0.5 mol/L for URE and 0.3 mol/L for GHC, because the resultant cryogels were very weak or not formed at all at a higher additive content. Contrary to this, when PVACG samples were prepared in DMSO, we were able to produce cryogels in the presence of much higher chaotrope concentrations (up to 4 mol/L for URE and 2 mol/L for GHC). A further increase in the concentrations of these additives was limited by their solubility in the DMSO solutions of PVA. In addition, along with such “novel” opportunities for the preparation of the PVACGs with increased content of URE and GHC, a very significant increase in the *E* and *T*_f_ values of the PVA/DMSO cryogels was unexpectedly observed. The peculiarities of these effects will be discussed below.

**Figure 1:**
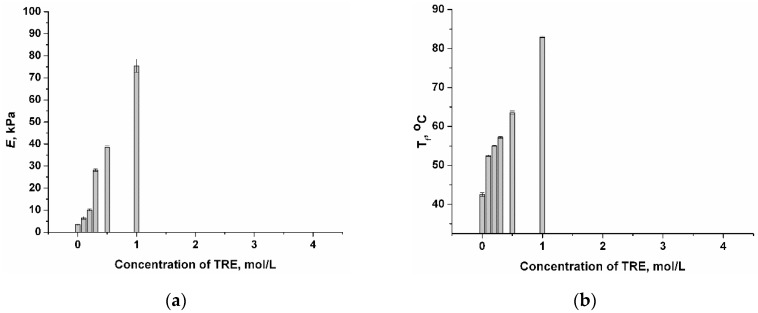
Dependences of the Young’s compression modulus (**a**) and fusion temperature (**b**) of the poly(vinyl alcohol) (PVA) cryogels formed in the DMSO medium in the presence of trehalose (THL) on the feed concentration of this additive ([PVA] = 100 g/L; freezing temperature: −21.6 °C or Δ*T* = −40 °C).

For the sake of comparison, we also fabricated PVACG samples in DMSO in the presence of THL as a kosmotropic agent. In this case, the feed PVA/DMSO/THL solutions were prepared only at kosmotrope concentrations of at most 1 mol/L because of a strong increase in the viscosity and limited THL solubility. The influence of the THL concentration on the properties of the resultant cryogels is illustrated in Figure 1.

When considering the data of Figure 1a,b, one can see a pronounced increase in the rigidity and heat endurance of the PVACGs formed in DMSO containing trehalose additives. As the THL concentration increases from 0 mol/L to 1 mol/L, a more than 22-fold increase in the Young’s modulus is observed (from 3.4 ± 0.3 kPa to 75.5 ± 3.0 kPa), and the fusion temperatures of the respective cryogels increase by nearly 43 °C from 42.5 ± 0.5 °C to 85.0 ± 2.2 °C. The values of these effects much exceed those caused by the THL additives upon the formation of PVACGs in aqueous systems where only a twofold increase in *E* was determined, and *T*_f_ increased by ~6 °C throughout the same range of THL concentrations [43]. In other words, the effect of this kosmotrope on the formation of PVA cryogels in DMSO was much stronger than in water. Nevertheless, this kosmotrope acted as the kosmotropes, i.e., at a qualitative level: without any anomalies.

**Figure 2:**
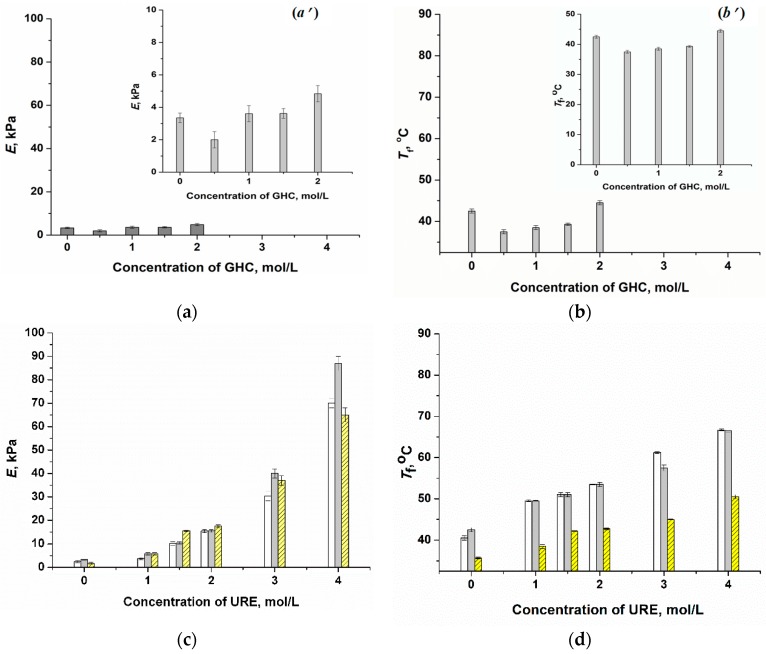
Dependences of the Young’s compression modulus (**a**,***a’***,**c**) and fusion temperature (**b**,***b’***,**d**) of the PVA cryogels formed in the DMSO medium in the presence of guanidine hydrochloride (GHC) (**a**,***a’***,**b**,***b’***) or URE (**c**,**d**) on the feed concentration of these additives ([PVA] = 100 g/L; freezing temperatures: −11.6 °C (Δ*T* = −30 °C)—white columns; −21.6 °C (Δ*T* = −40 °C)—grey columns; −31.6 °C (Δ*T* = −50 °C)—hatched columns).

However, anomalies in the properties of PVACGs (see above) unexpectedly appeared when chaotropic agents (GHC or URE) were introduced into PVA/DMSO feed solutions. It turned out that the chaotropes, especially URE, acted in a kosmotropic-like manner in relation to the cryotropic gelation of PVA in DMSO. These effects are shown in Figure 2 as the dependences of the Young’s modulus and gel fusion temperature on the chaotrope concentration. Although the experimental values obtained for the PVACGs containing GHC or URE differed considerably, the abscissa axes of the main plots in Figure 2 (as well as in Figure 1) are given in the same scale for the convenience of a juxtaposition of the trends observed. Since the effects caused by the GHC additives were significantly weaker than those caused by URE, the *E* and *T*_f_ values for the GHC-containing cryogels are additionally shown in Figure 2 as the insets *a’* and *b’*.

During these experiments, the attention was focused on the PVA–DMSO–URE systems, because of a wider range of “allowed” chaotrope concentrations in the feed solutions. In this case, the cryogel samples were prepared at three temperatures of freezing and subsequent incubation in a frozen state, viz., −11.6 °C, −21.6 °C, and −31.6 °C. Since the DMSO crystallization point (*T*_0_) is +18.4 °C [54], the aforementioned negative temperatures were respectively 30 degrees, 40 degrees, and 50 degrees below the *T*_0_. Cryogels in the PVA–DMSO–THL (Figure 1a,b) and PVA–DMSO–GHC systems (Figure 2a,b) were formed at −21.6 °C, that is, at −40 degrees in terms of the Δ*T* value [49], which equals the difference Δ*T* = –21.6 °C – 18.4 °C = –40 °C.

In a previous study, which dealt with the effects of chaotropes and kosmotropes on the cryotropic gelation of PVA in water [43], the gel-forming polymer of the same molecular weight characteristics was used. Its feed concentration was also 100 g/L, as in this work, where in contrast to the aqueous systems, it was possible to prepare cryogels in DMSO at twofold or threefold higher concentrations of the added GHC. Starting with GHC concentrations of about 1 mol/L, the resultant PVA–DMSO–GHC cryogels were characterized by somewhat higher rigidity and heat endurance than the additive-free samples with the same PVA content (Figure 2a,*a’*,b,*b’*). In particular, at [GHC] = 2 mol/L, the elasticity modulus of the cryogel was 4.8 ± 0.4 kPa versus 3.4 ± 0.3 kPa for the PVACG without additives, and the fusion temperatures of the corresponding samples were 44.5 ± 0.5 °C and 42.5 ± 0.5 °C. Thus, in contrast to aqueous systems, the GHC in DMSO did not affect the PVACG formation as a chaotrope, at least up to a concentration of 2 mol/L in the composition of the resultant PVACGs. At [GHC] = 1–2 mol/L, its influence can be characterized as the relatively weak “kosmotropic-like” effect. Interestingly, the dependences of *E* values on the GHC concentration (see Figure 2a,*a’*) had a slightly pronounced minimum at [GHC] = 0.5 mol/L, which was qualitatively very similar to the minima on analogous dependences reported in the study of the influence of kosmotropes on the strength of the PVACGs formed in aqueous media [43].

At the same time, urea additives exhibited surprisingly strong “reinforcing” effects. For instance, the elasticity modulus of the PVACGs formed in DMSO at [URE] = 2 mol/L increased (depending on the freezing temperature) to 15–18 kPa from 1.8–3.4 kPa for the additive-free samples (Figure 2c), thus demonstrating a nearly fourfold to tenfold increase. The “reinforcing” effect raised to nearly 12 to 21-fold at [URE] = 3 mol/L, and eventually to nearly 29 to 36-fold at [URE] = 4 mol/L, when the *E* values increased to 65–87 kPa (Figure 2c). At high URE content (3–4 mol/L), the most rigid PVACGs were formed at freezing temperature of −21.6 °C. At lower concentrations of the additive, the formation of somewhat stronger cryogels occurred upon freezing the feed solutions at a lower temperature of −31.6 °C. In fact, the growth in the gel strength induced by an increase in the URE concentration was accompanied by the increase in *T*_f_ values for the respective PVACG samples (Figure 2d). At [URE] = 4 mol/L, the resultant cryogels, depending on the freezing condition, could be fused at 51–67 °C (*cf*. 36–43 °C for the samples containing no additives). The higher fusion temperatures were inherent in the PVACGs formed at −11.6 °C and −21.6 °C as compared to the samples prepared by freezing at −31.6 °C. All of these results clearly demonstrated that urea acted as an efficient antichaotrope with respect to the freeze–thaw gelation of PVA in DMSO. More exactly, under these conditions, it exhibited a pronounced “kosmotropic-like” influence on the properties of the PVACGs that were formed in the presence of this non-ionic organic chaotropic (in water) agent.

**Figure 3:**
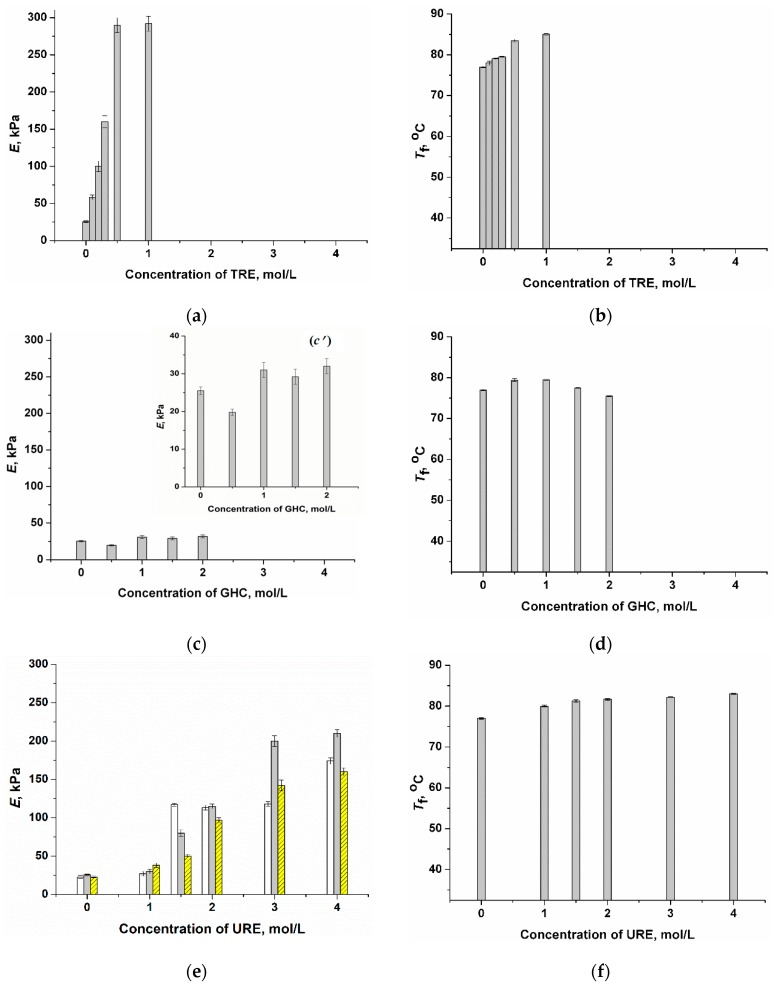
The Young’s compression modulus (**a**,**c**,***c’***,**e**) and the fusion temperature (**b**,**d**,**f**) of the secondary PVA cryogels that were initially formed in the DMSO medium in the presence of varied concentrations of THL (**a**,**b**), GHC (**c**,***c’***,**d**), or urea (URE) (**e**,**f**), and then rinsed with pure water (conditions of the cryogels preparation are the same as in the legend for Figure 2).

In this context, it was also of importance to reveal whether or not the trends that were observed for the variation of the physicochemical characteristics of PVACGs with an increase in the feed concentrations of THL, GHC, or URE will remain upon replacement of the organic liquid phase in the cryogels obtained by pure water. The results of these experiments are shown in Figure 3, which demonstrate a large increase in the *E* and *T*_f_ values for the ‘aqueous’ PVACGs. The *E*-modulus axes in Figure 3a,c,e span a wider range (up to 300 kPa) compared to the corresponding axes in Figure 1a and Figure 2a,c, since the water-rinsed, i.e., say, ‘secondary’ cryogels, were stronger than the ‘primary’ DMSO-containing PVACGs.

This effect turned out to be inherent not only in the cryogels formed with the addition of typical kosmotrope THL (*cf.* Figure 1a,b with Figure 3a,b); it was also observed for the samples formed in the presence of GHC (*cf.* Figure 2a,*a’*,b,*b’* with Figure 3c,*c’*,d, respectively) or URE (*cf.* Figure 2c,d with Figure 3e,f, respectively), as well as for the additive-free PVA cryogels (points of zero additive concentration in all of the diagrams). The juxtaposition of the rigidities of the ‘primary’ and ‘secondary’ PVACGs formed at −21.6 °C (Δ*T* = −40 °C) and at the highest concentrations of the additives used in this study shows that the *E* values increased from 76 kPa to 292 kPa (THL-caused effects), from 4.8 kPa to 32 kPa (GHC-caused effects), from 87 kPa to 210 kPa (URE-caused effects), and from 3.4 kPa to 25.5 kPa for the additive-free cryogels that were prepared using the same temperature regimes.

As to the variations of the *T*_f_ values, all of the water-rinsed PVACGs gained a higher heat endurance (Figure 3b,d,f) than the as-prepared samples of the corresponding DMSO-containing cryogels (Figure 1b and Figure 2b,d). First of all, for the additive-free PVACGs, a ~1.7-fold ‘jump’ of *T*_f_ values from 42.5 ± 0.5 °C to 77.0 ± 0.2 °C was observed. For the ‘secondary’ cryogels that were initially formed in the presence of an increasing amount (from 0 mol/L to 1 mol/L) of the kosmotropic agent THL, a gradual increase in *T*_f_ from 77 °C to 85 °C was recorded (Figure 3b). A similar trend took place for the heat endurance of the water-rinsed PVACGs prepared in the presence of URE (Figure 3f). The fusion temperature of the samples gradually grew from 77 °C ([URE] = 0) to ~80 °C ([URE] = 1 mol/L) and then to ~83 °C ([URE] = 4 mol/L) with the increasing URE concentration in the feed solutions of PVA in DMSO. The dependence of *T*_f_ on GHC for the ‘secondary’ PVACGs prepared with this additive was a bell-like type with a little pronounced maximum in the vicinity of [GHC] = 1 mol/L, and the fusion temperatures of these samples varied within the range of ~75–79 °C (Figure 3d). In any case, the significant increase in the *T*_f_ values upon going from the ‘primary’ DMSO-containing PVACGs to the ‘secondary’ water-rinsed cryogels was mainly due to the replacement of the thermodynamically good (for PVA) solvent DMSO by a poorer solvent (water) rather than to the type and amount of the low molecular weight additive as a component of the feed PVA solution to be gelled cryogenically. As a result of this replacement, a proportion of the polymer–solvent interactions is replaced by the polymer–polymer ones, thus causing an increase in the density of the 3D supramolecular network of the ‘secondary’ cryogels as compared with that of the ‘primary’ ones. This mechanism and the seven-day long (see Experimental) aging of the PVACGs in water caused a considerable increase in the heat endurance of the ‘secondary’ cryogels. Since the main type of the PVA–PVA interactions in aqueous media is the H-bonding [50,51,55] and the *T*_f_ values of the PVACGs, similarly to the majority of physical hydrogels, are the indicators of the amount of the thermocleavable hydrogen bonds [56,57], the de novo formed polymer–polymer links in the ‘secondary’ PVA cryogels should mainly be the H-bonds.

Of course, the increase in the network density should lead, and indeed led, to an increase in the rigidity of the ‘secondary’ PVACGs as compared to the ‘primary’ PVA cryogels. In this case, the basic character of the trends of the influence of the GHC and URE additives on the *E* values that was observed for the ‘primary’ PVACGs formed in DMSO (Figure 2a,*a’*,c) was also retained for the ‘secondary’ cryogels (Figure 3c,*c’*,e). However, in contrast to the fusion temperatures that were mainly controlled by the amount of the interchain H-bonds in the non-covalent PVA cryogels, the mechanical strength of the latter ones depends not only on the network density, but also on the macroporous morphology of such heterophase gel matrices. Therefore, we also studied the microstructure of the PVACGs of interest.

### 2.2. Influence of THL, GHC, and URE Additives as Components of the Feed PVA/DMSO Solutions on the Macroporous Morphology of the Resultant PVACGs

Optical microscopy studies were carried out using thin sections of water-rinsed PVA cryogels analogously to our previous study of PVACGs containing low molecular weight aliphatic alcohol additives [49]. The necessity of operating with the ‘secondary’ ‘aqueous’ cryogels (rather than the ‘primary’ DMSO-containing ones) was due to two main factors. First, DMSO itself and the DMSO-swollen polymeric walls of macropores in the ‘primary’ cryogels have very close refractive indices. Therefore, these PVACGs are almost transparent [46], and the peculiarities of their texture, i.e., pores and pore walls in the thin sections, are in fact indiscernible with an optical microscope. Second, it was found that the DMSO-containing PVA cryogels were practically not stained with the Congo red dye that was conventionally employed to contrast the thin sections of ‘aqueous’ PVACGs [9,43,49]. Taking these factors and the results of the *E* and *T*_f_ measurements into account, the following ‘secondary’ PVA cryogels were chosen for the microstructure studies:

- additive-free PVACG;
- cryogels prepared in the presence of 0.2 mol/L and 1 mol/L of THL (for the physicochemical characteristics, see Figure 1a,b, and Figure 3a,b);
- samples formed in the presence of 1 mol/L and 2 mol/L of GHC (moderate and highest concentrations of this additive; see Figure 2a,d and Figure 3c,d) and
- PVACGs prepared from the feed solutions containing 1 mol/L and 3 mol/L of URE (moderate and high concentrations of this substance; see Figure 2c,d and Figure 3e,f).

**Figure 4:**
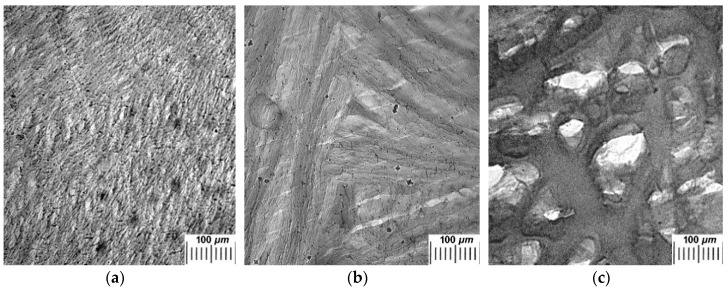
Micrographs of the Congo-red-stained thin sections of PVA cryogels (PVACGs) prepared without (**a**) and with the addition of THL in the concentrations of 0.2 mol/L (**b**) and 1.0 mol/L (**c**) (the physicochemical properties of these cryogel samples are shown in Figure 3).

**Figure 5:**
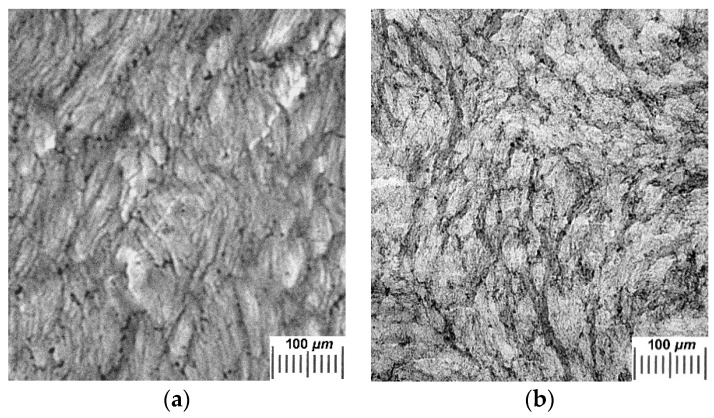

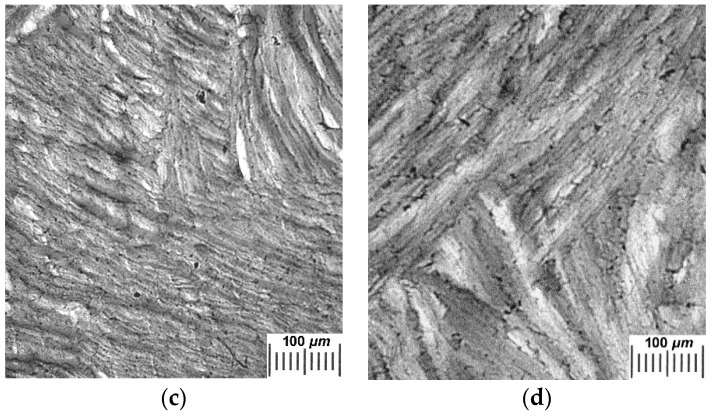
Micrographs of the Congo red-stained thin sections of PVACGs prepared with the addition of GHC in the concentrations of 1.0 mol/L (**a**) and 2.0 mol/L (**b**), and with the addition of URE in the concentrations of 1.0 mol/L (**c**) and 3.0 mol/L (**d**) (the physicochemical properties of these cryogel samples are shown in Figure 3).

The photomicrographs that were obtained are presented in Figure 4 and Figure 5 as the black-and-white images, where the dark areas are the polymeric phase (water-swollen pore walls stained with Congo red) and the light areas are the water-filled macropores. All of these PVAGGs were prepared from 100 g/L PVA solutions in DMSO under identical freezing–thawing conditions: freezing temperature of −21.6 °C (Δ*T* = −40 °C) and a heating rate of 0.03 °C/min upon the samples defrosting.

First of all, consider how the THL additive, the kosmotrope in both water [43] and DMSO, influences the microstructure of the PVACGs samples that were studied in this work (Figure 1 and Figure 3a,b). Even at a qualitative level, the images demonstrate clearly seen differences between the macroporous morphology of the additive-free and additive-containing samples (Figure 4). In particular, PVACG formed by the freeze–thaw gelation of the PVA solution in DMSO without additives has a relatively regular porous microstructure (Figure 4a). The pores in the sample mainly have anisometric shape, being 2–8 µm in the cross-section. A similar pattern was observed earlier for the additive-free PVACG that was formed by freezing a DMSO solution of the same polymer at a higher temperature (Δ*T* = −30 °C) [49]. Therefore, this macroporous morphology is obviously a characteristic feature of the PVA cryogels that were prepared in frozen DMSO and then rinsed with water. The character of the microstructure of PVACGs formed at comparable freezing conditions from the foreign additive-free aqueous solutions of this polymer is similar, but the pore size is somewhat smaller [42,43].

Already at a THL feed concentration of 0.2 mol/L corresponding to the weak influence of this kosmotrope on the gel strength (Figure 1a), the macroporous morphology of the resulting PVACG (Figure 4b) underwent a noticeable modification: the structural elements enlarged considerably and the cryogel texture became more heterogeneous and “rougher”. A further increase in the THL concentration up to 1.0 mol/L resulted in drastic alterations of the cryogel macroporous morphology with the formation of the “Swiss cheese-like” texture possessing the system of very large pores from ~50 to ~150 µm in size (Figure 4c). One can assume that this phenomenon is a consequence of the limited solubility of THL in DMSO. Since at room temperature, the THL concentration of 1 mol/L is close to the solubility limit of THL in DMSO, freezing the PVA–THL–DMSO feed solution should be accompanied by the partial precipitation of trehalose. Further formation of PVA cryotropic gel will seal up the precipitated particles in the gel bulk. After the system’s defrosting, the de novo dissolution of the THL particles, and then rinsing the sample with water, the surface tension forces will round the shape of the holes (large pores) thus formed. This means that the foreign solutes of limited solubility, such as THL in the PVA–DMSO solution, act upon the freeze–thaw-induced gelation of PVA as additional porogens along with the major porogens, that is, polycrystals of the frozen solvent [1,5,9,10,58,59,60,61]. If the proposed mechanism is in operation, small particles of THL precipitate can also serve as nucleating agents with respect to solvent crystallization similarly to the limited solubility of the low molecular weight additives described by Jiang et al. [62] for the case of the cryotropic gel formation of the aqueous PVA solutions.

As to the influence of GHC (Figure 5a,b) and URE (Figure 5c,d) on the character of the structural peculiarities of PVACGs prepared in DMSO, the changes observed more or less depend on the nature and concentration of the added low molecular weight solute. However, a trend that was common to these two systems was a decrease in the structural regularity as compared to the additive-free cryogel (Figure 4a). An increase in the GHC and URE concentrations caused a noticeable increase in the diversity of the pore shapes and sizes. However, in any case, the generation of such large pores as in the case of the PVACG formed in the presence of 1 mol/L of THL (Figure 4c) was not detected in our experiments. It should also be noted that the cryotropic gel formation of aqueous PVA solutions that contained moderate concentrations of chaotropes (URE ~0.3 M and ionic GHC ~0.2 M) resulted in the less perfect (more diffuse) microstructure of the cryogels as compared to the additive-free samples [43].

### 2.3. On Possible Mechanisms of the “Kosmotropic-Like” Influence of Organic Chaotropes on the Freeze–Thaw Gelation of PVA in DMSO

The influence of the GHC and URE additives on the physicochemical properties (Figure 2 and Figure 3) and macroporous morphology (Figure 5) of the PVA cryogels formed in DMSO (see above) causes an evident question regarding the possible reasons for the “kosmotropic-like” effects of the substances that exhibit pronounced chaotropic properties in water. To answer this question, we explored the rheological properties of the additive-free and URE-containing dilute (1–10 g/L) PVA solutions in water and DMSO. The dynamic viscosity of the 100 g/L polymer solutions in DMSO without and with URE (3 mol/L) was also measured (see Section 3). The systems that contained URE were selected, since it is this additive that exerted the most pronounced “kosmotropic-like” impact on the rigidity and heat endurance of the PVA cryogels that we have featured in this study. It was expected that the values of the intrinsic viscosity and the Huggins constant will allow one to reveal the influence of URE on the hydrodynamic characteristics of the PVA macromolecules in dilute polymer solutions and evaluate the affinity of PVA to these solvents for at least two temperatures. In turn, the temperature dependences of the viscosity of the moderately concentrated (in terms of the Hirai concept [63]) PVA–DMSO solutions in the absence and presence of URE allowed a rough evaluation of the PVA self-association energetics. The hydrodynamic and thermodynamic parameters of PVA in these solvents are listed in Table 1.

**Scheme 1:**
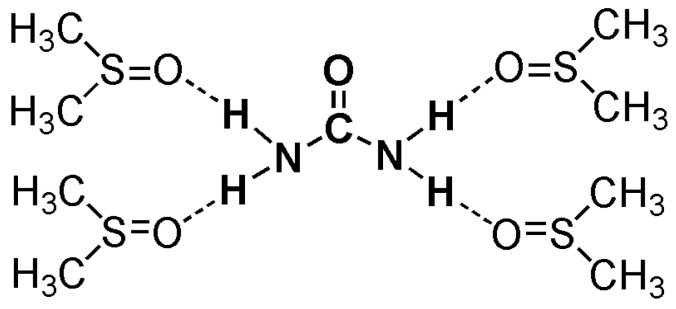
Possible scheme of URE complexation with DMSO.

The addition of URE to aqueous or DMSO solutions of PVA caused opposite changes in the intrinsic viscosity and Huggins constant. In the aqueous medium, the intrinsic viscosity increased from 0.6 dL/g to ~0.8 dL/g, but the *k’* value decreased from 2 to 0.8. This kind of change in the hydrodynamic characteristics of the polymer clearly points to the URE-caused improvement of the solvent quality [64] for PVA in water. In this case, the increase in [*η*] confirms better solvation and, as a consequence, a higher swelling extent of the PVA coils in the presence of urea, which leads to the larger hydrodynamic size of these coils as compared to the PVA coils in pure water. This effect is due to a well-known property of URE to compete efficiently for H-bonding in the polymer–polymer, polymer–water, and water–water interactions [44]. In these processes, urea, being an acceptor of protons, is able to disrupt the intramolecular and intermolecular hydrogen bonds between the OH groups of the PVA chains. At the same time, in the case of DMSO, the URE additive caused a decrease in the intrinsic viscosity of PVA from 1.7 dL/g in pure DMSO to ~1.4 dL/g in the 3-M URE–DMSO solution at 30 °C and correspondingly, from 1.3 dL/g to ~0.98 dL/g at 15 °C (see Table 1). Along with that, the Huggins constant at 30 °C remained almost unchanged, being somewhat decreased at 15 °C. These results point to the urea-induced decrease in the solvation ability of DMSO with respect to PVA. We believe that this effect can arise from specific reversible DMSO–URE interactions of the following kind (Scheme 1).

Here, an urea molecule can bind four DMSO molecules through the formation of H-bonds. Hence, at, e.g., [URE] = 3 mol/L, the concentration of the “H-bound” DMSO molecules should be around 12 mol/L. If the PVA concentration in the solution to be gelled by the freeze–thaw process is 100 g/L, the concentration of OH groups in this system is nearly 2.27 mol/L, i.e., the competition between the OH groups of the polymer and URE for binding with DMSO should shift the final result toward the formation of the URE–DMSO complexes rather than PVA solvation. In the dilute solutions used for the determination of [*η*], where the PVA concentration is much lower, a similar concentration prevalence of the URE–DMSO complexes is of course much higher. Therefore, urea causes the desolvation of the PVA macromolecules in DMSO, which leads to the partial collapse of the PVA coils, which manifests itself in a decrease in the intrinsic viscosity of the polymer. It should also be noted that this effect of urea on DMSO persists upon a decrease in temperature to 15 °C, which was the critical temperature of the DMSO supercooling in our experiments. This suggests that the poorer quality of DMSO as a solvent for PVA in the presence of urea is also retained at the temperatures of cryogenic treatment of the PVA–DMSO–URE solutions, thus being the main factor of the strengthening of the structure formation upon the freeze–thaw gelation of these systems.

Considering the influence of GHC additives on the results of the PVA cryotropic gel formation in DMSO (Figure 2a,b) from the same viewpoint, one can also explain the “weaker” GHC-induced effects compared to the URE-caused ones (Figure 2c,d). By analogy with urea, a guanidine molecule containing five potentially exchangeable protons can participate in the multipoint H-bonding with the DMSO molecules. However, GHC is the hydrochloride salt in which the joint effect of the delocalization of the electron density and protonation [65] somewhat deteriorates the ability of GHC to form H-bonds, thus interfering with the desolvation of PVA in DMSO, which becomes less efficient than in the case of URE. Therefore, the “kosmotropic-like” impact of ionic guanidine hydrochloride on the formation of PVA cryogel is less pronounced than in the case of the effects caused by the non-ionic urea additive.

We also determined the excess activation heat of viscosity (Δ*Q*^E^_η_) of the 100 g/L PVA solutions in pure DMSO and in the DMSO/3M-URE binary solvent. According to the theory by Hirai [63], this physical quantity is governed by the density and energetics of the physical knots of the temporary macromolecular network in a polymer solution:

(1) $$\Delta Q_{\eta }^{E}\sim \frac{M}{M_{c}}\Delta E$$

where *M* is the molecular weight of the polymer, *M_c_* is the average molecular weight of the subchains within the transient network, and Δ*E* is the dissociation energy of the network knots.

It is reasonable to suggest that the *M*/*M_c_* ratio is proportional to the reduced concentration of the polymer in the solution, *c*/c*, where c* = 1/[*η*]. In this case, the excess activation heat of viscosity is proportional to the product of the intrinsic viscosity by the dissociation energy of the network knots:

(2) $$\Delta Q_{\eta }^{E}\sim \left[\eta \right]\Delta E$$

or:

(3) $$\frac{\Delta Q_{\eta }^{E}}{\left[\eta \right]}\sim \Delta E$$

Thus, Formula (3) indicates that the ratio of the excess activation energy of the viscosity to the intrinsic viscosity of a polymer is a qualitative measure of the dissociation energetics of the physical knots of the transient macromolecular network in the polymer solution. From the data in Table 1, it follows that the values of this ratio for PVA solutions in DMSO and in the DMSO/3M-URE binary solvent coincide within the limits of the experimental errors of determination. Based on this fact, we can suggest a similar nature of the physical knots of the transient network of PVA macromolecules in both solvents. Most likely, these knots are formed due to the interactions of the poorly solvated segments of the polymer chains.

## 3. Experimental Section

### 3.1. Materials

The following compounds were used: poly(vinyl alcohol) (molecular weight of ca. 86 kDa, the deacetylation degree of 100%; Acros Organics, Morris Plains, NJ, USA), dimethyl sulfoxide (>99.8%; Komponent-Reaktiv, Moscow, Russian Federation), urea (“ultra pure” grade; Sigma, Ronkonkoma, NY, USA), guanidine hydrochloride (>99.5%; Helicon, Moscow, Russian Federation), trehalose (>98%; Panreac, Barcelona, Spain), the Congo red dye (Aldrich, St. Louis, MO, USA), as well as gelatine (photo quality), phenol (“analytically pure” grade), and glycerol (“analytically pure” grade) (all from Reakhim Co., Ekaterinburg, Russian Federation).

### 3.2. Preparation of Feed PVA Solutions

The dissolution of PVA in DMSO or in water was performed as described elsewhere [49]. Briefly, a known amount of dry polymer was dispersed in a calculated volume of DMSO to reach a PVA concentration of 100 g/L (for dynamic viscosity measurements and PVACGs preparation) or in the range of 1–10 g/L for the viscometry of dilute PVA solutions. The mixture was incubated for 18 h at room temperature for swelling of the polymer, and then, the system was heated for 1 h on a boiling water bath under stirring until the completion of PVA dissolution. When preparing the feed solutions that should contain an organic chaotrope or kosmotrope, the required amounts of additives were dissolved in the polymer solution at room temperature. The final solutions were sonicated for 20 min at room temperature in an UNITRA ultrasonic bath (Unitra, Poland) to remove air bubbles.

### 3.3. Viscometry of Dilute PVA Solutions

The viscosity of dilute PVA solutions in different solvents was measured with an AV-1 automatic Zimm viscometer (“Biopribor” Research and Production Association, Pushchino, Russian Federation) at a shear rate of 0.64 s^−1^ and at two temperatures of 15 °C and 30 °C. The standard error in the viscosity determination was less than ±0.03 cP. The solvents used were deionized water, a 3-M aqueous urea solution, neat DMSO, and a 3-M URE solution in DMSO. The dependencies of the specific viscosity (*η*_sp_) of the solutions on the PVA concentration (*c*) were used to determine the intrinsic viscosity ([*η*]) and the Huggins constant (*k’*) in terms of the Huggins equation [66] without its linearization:

(4) $$\eta_{\mathrm{sp}\;=\;}\left[\eta \right]c+k^{’}{\left[\eta \right]}^{2}c^{2}$$

Calculations were performed using the proprietary computer program Viscometry, which was developed at the A. N. Nesmeyanov Institute of Organoelement Compounds.

### 3.4. Viscometry of Moderately Concentrated PVA Solutions

The dynamic viscosity of the 100 g/L PVA solutions was measured over the temperature range of 15–30 °C at a constant shear rate of 1 s^−1^ using a Physica MCR-302 rheometer (Anton Paar, Graz, Austria) equipped with an automatic gap control system Tru-Gap™. The measuring plate–plate cell was 50 mm in diameter. The steady-state flow regime over the temperature range used was reached after about 5 s for all of the liquid systems under study. The temperature dependences of the relative viscosity of solutions were analyzed using the Arrhenius equation in the version by Hirai for the case of moderately concentrated polymer solutions [63]:

(5) $$\frac{\eta }{\eta_{0}}=\exp\left[\frac{\Delta Q_{\eta }^{E}}{RT}\right]$$

where η and η_0_ are the viscosities of the solution and the solvent, respectively; Δ*Q*^E^_η_ is the excess apparent activation heat of viscosity; R is the universal gas constant (8.324 J/mol·K), and *T* is the temperature (K). By definition, Δ*Q*^E^_η_ = Δ*Q*_η_ − Δ*Q*_η0_, where Δ*Q*_η_ and Δ*Q*_η0_ are the apparent activation heats of viscosity for the solution and the solvent, respectively.

### 3.5. Preparation of PVA Cryogels

Samples of PVA cryogels were prepared analogously to the earlier described techniques [43,49,58]. In these experiments, samples for physicomechanical tests were formed in sectional duralumin molds (inner diameter 15 mm, height 10 mm). To determine *T*_f_ values, cryogels were formed in transparent polyethylene test tubes (inner diameter 10 mm), 3-mL portions of the polymer solution were poured, and a stainless steel ball (diameter 3.5 mm, weight 0.275 ± 0.005 g) was placed on the bottom of each tube. The containers and the tubes were put into the chamber of an FP 45 HP precision programmable cryostat (Julabo, Seelbach, Germany), and the samples were frozen and incubated at a preset temperature for 12 h. Then, the temperature was raised to 20 °C at a rate of 0.03 °C/min controlled by the cryostat microprocessor.

### 3.6. Characterization of PVACG Samples

The compression Young’s modulus (*E*) and the fusion temperature (*T*_f_) of PVACG samples were evaluated in accordance with the protocols described elsewhere [43,49]. The *E* and *T*_f_ values were measured for three samples; the samples were examined in three to five independent experiments. The results obtained were averaged.

In brief, the *E* modulus of the PVACG samples was determined from the linear portion of the stress–strain dependence that was found using a TA-Plus automatic texture analyzer (Lloyd Instruments, West Sussex, UK) at a loading rate of 0.3 mm/min. The tests were performed until reaching a 30% deformation. The measurements were accomplished for both the PVACGs prepared in DMSO and for the samples in which the organic liquid phase (that is, DMSO and the dissolved solutes) was replaced by pure water. In the latter case, the cryogel samples were incubated for seven days at room temperature in glass beakers that each contained deionized water (100-fold excess relative to the volume of the PVACG sample); the water was replaced with a fresh portion every day. The fusion temperatures of the PVACG samples were measured by placing the tightly corked polyethylene tube containing cryogel with the stainless steel ball at the bottom upside down into the water bath. The bath temperature was increased at a rate of 0.4 °C/min. The gel fusion point was determined as the temperature when the ball fell down onto the stopper of the test tube after passing through the fused gel. These experiments were also carried out for both the PVACGs that were initially formed in DMSO and for the gel samples in which the organic phase was replaced by pure water using the same procedure as for the ‘aqueous’ cryogels used to measure their rigidity.

### 3.7. Macroporous Morphology of PVACG Samples

The macroporous morphology of the PVA cryogels was examined using an Eclipse 55i optical microscope (Nikon, Tokyo, Japan) equipped with a digital photocamera, as described earlier [49]. In the as-prepared PVACGs, DMSO was replaced by pure water prior to the fabrication of thin sections that were 10-µm thick and cut orthogonal to the axis of cylindrical samples using a SM-1900 cryomicrotome (Leica, Wetzlar, Germany). Each section was placed on the microscope glass, which was then immersed into a 1% aqueous solution of Congo red for staining for 10 s, and then rinsed with pure water. The excess of liquid was removed with a filter paper. Then, the section was poured with a drop of fixing solution (solution of 1 g of gelatin in 12 mL of 50% aqueous glycerol and 0.2 g of phenol as a bacteriostatic agent) and sealed with a cover glass. Prior to the studies, the samples were stored in a closed vessel at 4 °C.

## 4. Conclusions

Urea and guanidine hydrochloride are well-known chaotropes that efficiently suppress H-bonding in aqueous media [44]. This property of URE and GHC is widely used for various purposes when working with natural and synthetic water-compatible polymers [67]. In the case of the freeze–thaw-induced gelation of PVA, where intermolecular H-bonding is the key process that is responsible for the interchain linking [1,9,10,37,38,39], the introduction of these chaotropes into the feed aqueous polymer solution causes partial or complete inhibition of the PVACGs formation depending on the URE or GHC concentration [43]. However, it unexpectedly turned out that in the case of the PVA cryotropic gel formation in DMSO, the URE and GHC additives caused effects that were opposite to those observed in water, i.e., the formation of the PVACGs was unexpectedly strengthened rather than inhibited [53]. Systematic study, which was the task of this work, showed that such “kosmotropic-like” effects were more pronounced for the PVACGs formed in DMSO in the presence of URE rather than GHC additives, with the effects being concentration-dependent. In particular, at [URE] = 2 mol/L, the rigidity of the resulting cryogel increased four to 10-fold depending on the freezing temperature compared to the additive-free PVACGs that were formed under identical freeze–thaw processing conditions. At [URE] = 4 mol/L, the reinforcing effect was nearly 29 to 36-fold. The growth of the gel strength was accompanied by a considerable increase in the heat endurance of the samples. It was also shown that the additives of URE and GHC caused significant changes in the macroporous morphology of the cryogels. The commonly observed trend was a decrease in the structural regularity of the additive-containing samples as compared to the additive-free PVACGs. Viscosity measurements performed for the dilute and moderately concentrated URE-free and URE-containing solutions of PVA in DMSO allowed one to reveal the consistent changes in the intrinsic viscosity, Huggins constant, and the excess activation heat of the viscosity caused by the additives. These data evidently point to the urea-induced decrease in the solvation ability of DMSO with respect to PVA. As a result, this effect can be the key factor that is responsible for the strengthening of the structure formation upon the freeze–thaw gelation of this polymer in DMSO additionally containing the additives such as urea, which is capable of competing with PVA for the solvent. No doubt, in order to elucidate the fine molecular mechanisms of the interactions of PVA–DMSO–chaotropes in detail, some additional studies are required. In this regard, we intend to explore such systems using different spectroscopy methods, NMR, in particular.

Finally, it is worth noting that the possibility of preparing very strong PVA cryogels that have the Young’ modulus in the vicinity of 0.2–0.3 MPa (Figure 3a,e) could be of certain applied interest. For instance, a rather evident field of application of such PVACGs is the elaboration of artificial cartilages [16,18,19,20]. The procedure, which allows reaching such a rigidity level for the resultant PVA cryogels, is not complicated, since it only requires using the DMSO-soluble kosmotropic-like additives and, after a single freeze–thaw cycle, rinsing the resultant cryogels with water in order to replace the organic dispersive phase by the aqueous one. Therefore, it is not necessary to operate with very viscous high-concentrated PVA feed solutions or employ multiple freeze–thaw cycling, which are the approaches that are frequently used for increasing the hardness of various PVACGs [1,2,8,9,10,11,18,58,59].

## Figures and Tables

**Table 1:** Hydrodynamic parameters of PVA in different solvents ^a)^.

Solvent	30 °C		15 °C		Δ*Q*^E^_η_	Δ*Q*^E^_η_/[*η*]
	[*η*]	*k’*	[*η*]	*k’*		
Water	0.60 ± 0.03	2.0 ± 0.3	-	-	-	-
Water-3 M URE	0.79 ± 0.03	0.8 ± 0.1	-	-	-	-
DMSO	1.70 ± 0.10	0.4 ± 0.1	1.30 ± 0.10	1.1 ± 0.4	29.4 ± 0.9	17 ± 2
DMSO-3 M URE	1.36 ± 0.03	0.44 ± 0.05	0.98 ± 0.05	0.8 ± 0.2	26.0 ± 0.9	19 ± 1

^a)^ [*η*] is the intrinsic viscosity (dL/g); *k’* is the Huggins constant; Δ*Q*^E^_η_ = *Q*_η_ − *Q*_η__0_ is the excess apparent activation heat of viscosity (kJ/mol); *Q*_η_ and *Q*_η__0_ are the apparent activation heats of viscosity for the polymer solution and solvent, respectively.
